# Introducing an Austrian backpack in Spain

**DOI:** 10.1007/s13209-022-00263-x

**Published:** 2022-04-18

**Authors:** João Brogueira de Sousa, Julián Díaz-Saavedra, Ramon Marimon

**Affiliations:** 1 Universidade Nova de Lisboa, Lisbon, Portugal; 2 Universidad de Granada, Granada, Spain; 3 Universitat Pompeu Fabra, Barcelona School of Economics, Barcelona, Spain; 4 CEPR and NBER, European University Institute, Fiesole, Italy

**Keywords:** Computable general equilibrium, Welfare state, Social security reform, Retirement, C68, H55, J26

## Abstract

In an overlapping generations economy with incomplete insurance markets, the introduction of an employment fund—akin to the one introduced in Austria in 2003, also known as ‘Austrian backpack’—can enhance production efficiency and social welfare. It complements the two classical systems of public insurance: pay-as-you-go (PAYG) pensions and unemployment insurance (UI). We show this in a calibrated dynamic general equilibrium model with heterogeneous agents of the Spanish economy in 2018. A ‘backpack’  (BP) employment fund is an individual (across jobs) transferable fund, which earns a market interest rate as a return and is financed with a payroll tax (a BP tax). The worker can use the fund while unemployed or retired. Upon retirement, backpack savings can be converted into an (actuarially fair) retirement pension. To complement the existing PAYG pension and UI systems with a welfare maximizing 6% BP tax would raise welfare by 0.96% of average consumption at the new steady state, if we model Spain as an open economy. As a closed economy, there are important general equilibrium effects, and as a result, the social value of introducing the backpack is substantially greater: 16.14%, with a BP tax of 18%. In both economies, the annuity retirement option is an important component of the welfare gains.

## Introduction

The reform of twentieth-century public insurance systems is an ongoing issue which the twenty-first-century crises—financial, euro-debt and COVID-19—has only exacerbated. It has been the focus of extensive research in economics and other social sciences, most of it analyzing the possible reform of separate specific systems: either the pay-as-you-go pension system or the unemployment insurance system (UI). However, there is an interesting reform that, in our opinion, deserves further analysis: this is the introduction of an employment fund, also known as the ‘Austrian backpack’  as a social protection policy.

The basic features of a ‘backpack’  (BP) employment fund are: it is a fund owned by the employee which accumulates, with a basic payroll tax, while working; it is transferable across jobs and can be used while unemployed and as a pension fund; it earns a market interest rate (i.e., it can be privately managed), but there may be restrictions in its use (e.g., additional individual contributions may be restricted and the worker may only be able to use it is unemployed, inactive, or retired). While different forms of private employment funds are not a novelty, in most advanced countries such funds are not part of the public social protection policy. Similarly, some countries have tax exemptions for limited amounts of savings placed in private pension funds.^1^ BP funds have in common with these private employment and pension funds their portability and favorable tax treatment. In contrast, the BP is designed to be comprehensive (mandatory savings) and targeted to complement public unemployment insurance benefits and retirement pensions; that is, the BP is a public social protection policy.

The ‘Austrian backpack’  was introduced in 2003 as part of a broader labor reform in Austria. In particular, it was the socially agreed exchange for the gradual phase out of severance payments. The ‘backpack tax’  of 1.5% was set according to this trade-off. Kettemann et al. (2017) have shown that this reform increased job creation and lowered unemployment, although the main effect of the reform came from the elimination of severance payments. Based on this experience and the extensive literature on the Spanish dual labor market, the Bank of Spain and OECD, among others, have proposed the introduction of a Backpack system in Spain, as replacement to the existing severance payment system.^2^ The welfare gains from introducing such reform in Spain are likely to be larger than in Austria, since it will also help to break the existing duality between contracts with and without severance payments. Nevertheless, a backpack designed exclusively for this reform is likely to be relatively small, as in Austria. It is worth studying the role and optimal size of the BP contract as a complement to existing social protection and labor market policies, which is the objective and contribution of this paper. We consider an enhanced version of the backpack contract: while unemployed workers can still decide how much to withdraw from their BP fund while unemployed (as in the Austrian case), upon retirement, BP assets can be converted in a (actuarially fair) pension payment.

Our paper is related to the literature on unemployment insurance and retirement pension systems. On the UI literature, it is related to the studies on unemployment insurance savings accounts (Feldstein (2005); Feldstein and Altman (2007) and Setty (2017)) as complements or substitutes of existing tax transfer UI systems. It is also related to the literature on reform of unfunded retirement pension systems staring with the pioneer work of Auerbach and Kotlikoff (1987); Kotlikoff et al. (1999) and De Nardi et al. (1999), and more recently to the adverse effects of aging on PAYG pension systems, in particular the Spanish case ( Díaz-Giménez and Díaz-Saavedra (2009, 2017)). Our paper complements this literature by studying a fully funded insurance system that complements (and can potentially substitute) both unemployment insurance and pay-as-you-go retirement pensions, using a calibrated model of the Spanish economy which has had historically high unemployment rates and a demographic aging process that threatens the sustainability of the social security system.

Our work builds directly on, and integrates, two models: the model of Díaz-Giménez and Díaz-Saavedra (2009) and Díaz-Giménez and Díaz-Saavedra (2017), developed to study pension system reforms in Spain using overlapping generations general equilibrium models, and the model with job creation and destruction with search frictions and three employment states (employed, unemployed, and inactive) of Krusell et al. (2011), further developed in Ábrahám et al. (2021) to study unemployment insurance reforms in Europe. In the model economy, agents—which we refer to as households—can differ by their age, education, and labor productivity, and they decide how much to save and consume, as well as their employment status, which also depends on the rates of job creation and destruction. In our benchmark economy, households can insure against their idiosyncratic risks privately, through their savings, but they cannot borrow. There is public unemployment insurance and a pay-as-you-go pension system, both financed with payroll taxes. The government must balance the yearly budget. The model is calibrated to the Spanish economy in 2018, as an open economy, taking into account existing tax and transfer systems.

We compare steady states of the economy under different public insurance systems and find a welfare maximizing ‘backpack tax’  at 6%, four times the size the one introduced in Austria. We compute the new steady state under the backpack system and compare it with the status quo economy. The effects within the economy are relatively small in terms of macroeconomic aggregates since prices (interest rates and wages) do not change under an open economy assumption. Nevertheless, there are significant variations on households’ decisions: substitution of private for backpack savings with an overall increase of asset holdings; consumption is smoother during unemployment spells, and when BP savings are annuitized, smooth and substantially higher upon retirement. Labor market participation is higher, with a substantial reduction of inactive people, and retirement is slightly delayed. In sum, there is a small increase on employment, hours worked, productivity and, therefore, output. We assess the welfare gains by the lifetime consumption equivalent variation of the average household entering the economy at 20. The introduction of the backpack represents a 0.96% increase in welfare. There is a small increase in income inequality and larger one on wealth inequality since, at the individual level, more productive agents accumulate a larger fraction of assets.

We also calibrate and analyze Spain as a closed economy, as an alternative benchmark, to understand how a non-fully open economy will react to the introduction of the backpack. Aggregate general equilibrium effects are significant, with a substantial reduction of the economy’s interest rate and increase in wages as the Backpack system is introduced. As a result, the effect on households’ decisions is exacerbated, which translates into substantial aggregate effects and welfare gains. The introduction of the backpack in the closed economy enhances efficiency more than in the open economy (allocation of resources and productivity), and it also exacerbates a distortion on consumption plans that the backpack generates: a jump on consumption at retirement, when the backpack assets become fully available for consumption.

The comparison of the open and closed economies is also relevant when we assess the role of the different aspects of the backpack system (forced savings while working, consumption smoothing during unemployment and safe annuity payments after retirement). We find that annuity payments are an important feature of the backpack contract. As we show in our simulations, without the option to annuitize BP savings there are no welfare gains from introducing the backpack system in an open economy, while there are still substantial gains in the closed economy.

We also show (in Appendix) that a reform consisting on replacing the existing unemployment system for the backpack is not welfare improving, because younger generations do not accumulate backpack assets fast enough as to provide enough insurance during unemployment spells at the beginning of the career. In other words, the backpack tax would be too high in early ages, decreasing after-tax labor income, and the gains in insurance provision at older ages, during retirement, are not enough to compensate this distortion.

The next section presents our model economy, Sect. 3 presents the calibration to the Spanish data, Sects. 4 and 5 present the results for the open and closed economy models, Sect. 6 presents the role of the annuity payments in the backpack contract and Sect. 7 concludes, with additional supporting material and results conveyed in Appendix.

## The model economy

This section presents the model economy. We study an overlapping generations economy with heterogeneous households, a representative firm, and a government. We use the same model in related work in Brogueira de Sousa et al. (2021). Although we calibrate the model economy to Spanish data, here we describe an economy that features a backpack system, in addition to the PAYG pension system. For the calibration procedure, we set one parameter in the model to zero—the Backpack contribution rate—and match the model to Spanish data. In the policy reform exercise, we compare the calibrated model to alternative Backpack economies.

Time is discrete and runs forever, and each time period represents one calendar year. All model quantities depend on calendar time *t*, but we omit this dependence since we focus on steady-state equilibria. In a steady state, as defined below, all aggregate variables and individual policy functions are constant with respect to calendar time. We begin with a description of household heterogeneity.

### The households

Households in our economy are heterogeneous and differ in their age, $j \in J$; in their education, $h \in H$; in their productivity level $z \in {\mathcal {Z}}$; in their labor market status $s \in S$; in their private assets, $a \in A$; and in their backpack savings, $b \in B$. Sets *J*, *H*, ${\mathcal {Z}}$, *S*, *A*, and *B* are all finite sets and we use $\mu _{j,h,z,s,a,b}$ to denote the measure of households of type (*j*, *h*, *z*, *s*, *a*, *b*). They also differ in their claims to different social insurance systems: unemployment benefits *UB*, retirement PAYG pensions *P*, and government transfers *TR*. We think of a household in our model as a single individual, even though we use the two terms interchangeably. To calibrate the model, we use individual data of persons older than 20.

#### Age

Individuals enter the economy at age 20, the duration of their lifetimes is random, and they exit the economy at age $T=100$ at the latest. Therefore, $J=\{20,21,...,100\}$. The parameter $\psi _j$ denotes the conditional probability of surviving from age *j* to age $j+1$. The notation makes explicit that the exogenous probabilities depend on age *j*, but not on education or other factors.

#### Education

Households can either be high school dropouts with $h=1$, high school graduates who have not completed college $h=2$, or college graduates denoted $h=3$. Therefore, $H=\{1,2,3\}$. A household’s education level is exogenous and determined forever at the age of 20.

#### Labor market productivity

Individuals receive an endowment of efficiency labor units every period. This endowment has two components: a deterministic component, denoted $\epsilon _{h,j}$ and a stochastic component, denoted by *z*. The deterministic component depends on the household’s age and education, and we use it to characterize the life cycle profiles of earnings. The stochastic component is independently and identically distributed across households, and we use it to generate earnings and income dispersion in the economy. This component does not depend on the age or the education of the households, and we assume that it follows a first order, finite state, Markov chain with conditional transition probabilities given by $\Gamma$:

1 $$\begin{aligned} \Gamma \left[ z'|z\right] = \text {Pr}\left\{ z_{j+1}=z'|z_j=z\right\} ,\;\text {with}\;z,z'\in Z. \end{aligned}$$

Every period agents receive a new realization of *z*. Total labor productivity is then given by $\epsilon _{h,j}z$. A worker who supplies *l* hours of labor has gross labor earnings *y* given by:

2 $$\begin{aligned} y = \omega \epsilon _{h,j} z l, \end{aligned}$$

where the economy-wide wage rate $\omega$.

#### Labor market status

In the model, an agent is either employed, unemployed, non-active, or retired. Among the unemployed, there are individuals who are eligible to receive unemployment benefits and access their backpack savings (workers who have recently been laid off), and others who are not eligible (either because eligibility expired, or because they quit work). Worker decide when to retire (with a minimum retirement age), leaving the labor force permanently once they do. Upon entering the economy, individuals randomly draw a job opportunity and then decide to work or not during the first period. Similarly, in subsequent years the labor market status evolves according to both optimal work and job search decisions (described below), and exogenous job separation and job finding probabilities.

#### Employed

An individual with a job in the beginning of the period, and who decides to work, is employed in that period and his labor market status is denoted by $s=e$. An employed worker provides labor services and receives a salary that depends on his efficiency labor units and hours worked. He faces a probability of losing the job at the end of the period, denoted $\sigma _{j}$. This probability is age dependent, and we use it to generate the observed labor market flows between employment and non-employment states within age cohorts.

#### Unemployed

An agent may not have a job opportunity at the beginning of a period, because he lost his job last period, because he quit his job, or because he was unemployed last period and did not find (or did not accept) a new job offer. Without a job, households may actively search for a job offer next period. If they do actively search we label them as unemployed. Unemployed agents who have lost a job are eligible for unemployment benefits and to use accumulated backpack savings (we refer to them as *unemployed eligible*, with $s=ue$). A formal description of eligibility criteria is given below. Agents who have quit work are not eligible for unemployment compensation (we often refer to this group as *unemployed non-eligible*, $s=un$). Active job searchers receive a job offer at the end of the period with probability $\lambda ^{\mathbf{u }}_{j}$. The probabilities are again age dependent, and we use it to generate the observed labor market flows between unemployment and employment.

#### Non-active

Agents without a job and who do not actively search for a new one are labeled non-active, with $s=n$. Those agents are not eligible for unemployment benefits nor to collect backpack savings, and receive a job offer for next period with a lower probability than an unemployed agent, $\lambda ^{\mathbf{n }}_{j}<\lambda ^{\mathbf{u }}_{j}$. This probability is also age dependent, and we use it to generate the observed labor market flows between non-activity and employment.

#### Retirees

In our model, workers optimally decide whether to retire and leave the labor force (with a minimum retirement age). They take this decision after observing their current labor productivity. If they decide to retire, $s=r$, they lose the endowment of labor efficiency units for ever and exit the labor market. Depending on the retirement savings systems in place, they receive PAYG retirement pension payments and may receive additionally a backpack annuity payment.

#### Private assets

Households in our model economy endogenously differ in their asset holdings, which are constrained to being nonnegative. The absence of insurance markets give the households a precautionary motive to save. They do so by accumulating real assets which take the form of productive capital, denoted $a\in A$. Different retirement pension systems affect, among others, the agents’ private savings decisions.^3^

#### Backpack assets

Workers accumulate backpack savings while they work. These savings result from a mandatory contribution out of workers’ salaries, and are invested in productive capital and earn the real rate of return in the international capital market. When workers lose a job, they can access their accumulated savings and decide how much to keep in their individual accounts or how much to use, while out of work, to finance consumption. A formal description of the decision problem is given below. At retirement, backpack assets are converted into retirement pension payments (an actuarially fair life annuity).

Households derive utility from consumption, and disutility from labor and the search effort. Labor is decided both at the extensive and intensive margins, while search is a discrete choice. The period utility is described by a utility flow from consumption and the utility cost of time allocated to market work and to job search. Non-active and retired agents dedicate all the time endowment to leisure consumption. Accordingly, lifetime utility is given by

3 $$\begin{aligned} {\mathbb {E}} \sum _{j=20}^{100} \beta ^{j-20}\psi _{j} \Big [ u(c_j, l_j) - \gamma e_j \Big ], \end{aligned}$$

where $\beta$ is a time discount factor, *u* satisfies standard assumptions, $c_j$ is consumption and $l_j$ is labor supply, and $\gamma$ represents a job search utility cost. $l_j$ can take values between 0 and 1, while $e_j$ equals 1 in periods of active job search and is zero otherwise. Survival probabilities $\psi _{j}$ determine average life expectancy in the economy, a central object in our analysis.^4^

At the beginning of each period, *z*, households’ stochastic productivity component, is realized.^5^ When entering the economy (at age 20) agents additionally learn their education level and draw a job opportunity, that they can either accept of reject. For older households, if they start a period with a job opportunity, they decide whether to work and if so, by how much. If they lost their job or decided not to work in the previous period, they choose whether to search for a new job or not. Depending on these decisions, individuals then spend the period working, unemployed or inactive. Wages and unemployment benefits are received, and decisions on consumption and savings are taken. At the end of the period, workers observe the job separation shock, and unemployed or inactive learn if they found a job for next period. Households can choose to retire at the beginning of the period, and once they do they leave the labor market permanently.

### Technology

*The firm.* In our model economy, there is a representative firm. Aggregate output depends on aggregate capital, *K*, and on the aggregate labor input, *L*, through a constant returns to scale, Cobb–Douglas, aggregate production function of the form

4 $$\begin{aligned} Y = K^\theta L^{1-\theta }. \end{aligned}$$

Factor and product markets are perfectly competitive and the capital stock depreciates geometrically at a constant rate, $\delta$. The firm rents capital in the international capital market at an interest rate *r* and hires workers in the domestic market at a wage rate $\omega$ per efficiency unit of labor.

*Insurance markets.* An important feature of the model is that there are no insurance markets for the stochastic component of the endowment shock, for unemployment risk, or survival risk.

### Backpack system

The BP economy features a fully funded employment fund, financed by individual worker contributions. Workers may choose to use all or a fraction of the BP savings during periods of involuntary unemployment. Every individual enters the economy without backpack claims. For every period of employment, a worker sees a fraction $\tau _b$ of his gross labor earnings deducted and invested into a personal savings account, which is remunerated at the capital market rate of return, *r*. If $b_t$ is the level of backpack assets at the beginning of an employment period, then next period’s backpack evolves according to:

5 $$\begin{aligned} b_{t+1} = \tau _b y + (1+r(1-\tau _k))b_t, \end{aligned}$$

with $\tau _k$ being the capital income tax rate. When a worker loses his job, his backpack assets can be allocated to finance consumption (present or future, as he can choose to save the backpack assets). Next period’s backpack assets become a choice variable for the involuntary unemployed. In contrast, if a worker chooses to quit his job while still in the labor force, he keeps the backpack but cannot withdraw. In that period, the backpack evolves according to

6 $$\begin{aligned} b_{t+1}= (1+r(1-\tau _k))b_t. \end{aligned}$$

Upon retirement, backpack assets can be used to buy a lifetime annuity or added to private savings. If the worker decides retire at age *R* and allocate *b* amount of BP savings to the purchase of the annuity contract, he receives in return:

7 $$\begin{aligned} p^B(b) = b\left[ 1 + \sum _{t=1}^{T-R}\frac{\prod _{i=0}^t\psi _{R+i}}{(1+r)^t}\right] ^{-1}. \end{aligned}$$

The aggregate amount of backpack assets is invested in the capital market and adds to the stock of productive capital available in the economy. Since this is an individual, fully funded system, the aggregate amount of BP assets used to purchase annuity contracts equals the total amount of annuity payments received by retirees. Hence, we do not include it in the social security budget equation, shown below.

### Pay-as-you-go system

The PAYG system is an unfunded defined contribution pension system, where pension payments mostly depend on individual workers’ history of salaries, among other factors. In the model, pension payments depend on average earnings during the $N_b$ years prior to retirement. In Spain, as in many other countries where a PAYG system exists, there is a minimum retirement age after which worker can decide to retire. We denote it by $R_0$. In order to capture the heterogeneity in pension payments that arises from different lifetime earnings histories, but at the same time reduce the dimensionality of the problem, we model pension payments that differ for each educational group (instead of each individual). Specifically, pension payments for retirees of educational group *h* are:

8 $$\begin{aligned} p^S_h=p_r{\bar{y}}^S_{h}, \end{aligned}$$

where ${\bar{y}}^S_{h}$ is the average earnings of households in educational group *h* during the last $N_{b}$ years before the retirement age, $R_0$, and $p_r$ is a replacement rate. ${\bar{y}}^S_{h}$ is computed as:

9 $$\begin{aligned} {\bar{y}}^S_{h} = \frac{1}{N_{b}}\sum _{j=R_0-N_{b}}^{R_0-1} {\bar{y}}_{j,h} \end{aligned}$$

where ${\bar{y}}_{j,h}$ is the average gross labor earnings of workers aged *j* and with education *h*. We assume that there are no early retirement penalties, nor minimum or maximum pensions.

### The government

Here we describe the government programs other than retirement pensions, discussed above, and the government and social security budgets that we model separately.

*Unemployment benefits.* The government taxes workers and provides unemployment benefits to the unemployed. Eligibility for unemployment benefits—denoted $\mathbbm {1}_{UB}=1$, below—is conditional on: (i) having lost a job (i.e., a job separation) and not having started a new job yet, (ii) on actively searching for a job, and (iii) having been unemployed for less than a given number of periods, ${\bar{d}}$. Eligibility expires when one of the conditions is not met, and non-eligibility is an absorbing state. Eligible agents receive unemployment benefits given by $u_b = b_{0} {\bar{y}}_{j,h}$, where $b_0\in (0,1)$ is a replacement rate and ${\bar{y}}_{j,h}$ is the average labor earnings of workers in age group *j* and with education *h*. Unemployment benefits are financed with payroll taxes, described below.

*Other transfers* Households below an income level $y<\overline{t_{r}}$ receive a transfer from the government, denoted *TR*. Eligibility for transfers is conditional on income only and denoted by $\mathbbm {1}_{TR}=1$. Eligible households receive an amount $t_r=b_{1}\overline{t_{r}}$.

We model the government budget restriction with two separate identities. Unemployment benefits and PAYG pensions are financed with payroll taxes and form the social security budget. Other government expenditures and revenues form the government budget.^6^

The government taxes capital income, household income and consumption, and it confiscates (part of the) unintentional bequests. It uses its revenues to finance an exogenous flow of public consumption and debt, and to make transfers to low-income households. In addition, the government provides unemployment benefits and runs a PAYG pension system.

The government budget constraint is then:

10 $$\begin{aligned} G_t+T_{r,t}+D_{t+1}&= T_{k,t}+T_{y,t}+T_{c,t}+E_t+(1+r)D_t, \end{aligned}$$

11 $$\begin{aligned} U_{b,t} + P_t&= T_{p,t}, \end{aligned}$$

where $G_t$ denotes government consumption, $T_{r,t}$ denotes government transfers, $T_{k,t}$, $T_{y,t}$, and $T_{c,t}$, denote the revenues collected with the capital income tax, the household income tax, and the consumption tax, and $E_t$ denotes unintentional bequests taxed by the government. $U_{b,t}$ denotes unemployment benefits, $P_t$ denotes pension payments in period *t* and $T_{p,t}$ denotes revenues collected with the payroll tax. In the remaining of the paper, we assume that the level of public debt is fixed at the baseline calibration year level, $D_{t+1}=D_t$.

*Capital income taxes* Capital income taxes are given by $\tau _k y_k$, where $\tau _k$ is the tax rate on gross capital income $y_k = r a$. *a* denotes households’ capital holdings and *r* the economy rate of return on capital.

*Payroll taxes* Payroll taxes are proportional to before-tax labor earnings: $\tau _p y$.

*Backpack taxes* Similarly, contributions to accumulate assets in the individual Backpack account are given by: $\tau _b y$.

*Consumption taxes* Similarly, consumption taxes are simply $\tau _{c}c$, where $\tau _c$ is the consumption tax rate and *c* is consumption.

*Income taxes* We assume a simplified income tax formula according to which the income tax is proportional to the income level: $\tau _y {\hat{y}}$, where $\tau _y$ is a tax rate parameter and ${\hat{y}}$ is the tax base. The income tax base depends on the employment status. If a household is employed:

12 $$\begin{aligned} {\hat{y}}= (1-(\tau _p +\tau _b))y + r(1-\tau _k)a. \end{aligned}$$

For the unemployed and non-active agents,

13 $$\begin{aligned} {\hat{y}}= r(1-\tau _k)a, \end{aligned}$$

and for a retired household:

14 $$\begin{aligned} {\hat{y}}= r(1-\tau _k)a + p^S_h. \end{aligned}$$

### Individual decision problem

We describe the problem in the *BP* economy, i.e., a steady-state economy with a Backpack system and a PAYG pension system. The households’ problem is described recursively. To simplify the notation, we omit the dependence of the value functions on the state variables age, education, private savings, backpack savings, and unemployment spell duration.

We first state the decision problem of a worker at the beginning of the period after the job acceptance decision was taken. Given the value functions, we define below the job acceptance and retirement decisions. An individual who is currently employed decides how much to consume *c*, save $a'$, and work $l\in \left[ 0,1\right]$, according to the following optimization problem:

15 $$\begin{aligned} W = \max _{c,l,a'} \Bigg \{ u(c, l) + \beta {\mathbb {E}} \Big [ (1-\sigma _j) J + \sigma _j U \Big ] \Bigg \} \end{aligned}$$

subject to:

16 $$\begin{aligned} (1+\tau _c)c + a' + \tau _y{\hat{y}} + (\tau _p+\tau _b)y \le (1+r(1-\tau _k))a + y + TR(y) , \end{aligned}$$

the backpack law of motion,

17 $$\begin{aligned} b' = \tau _b y + (1+r(1-\tau _k))b, \end{aligned}$$

and a non-borrowing constraint:

18 $$\begin{aligned} a' \ge 0. \end{aligned}$$

Gross labor income is $y = \omega \epsilon z l$, income tax base ${\hat{y}} = (1-\tau _p-\tau _b)y + r(1-\tau _k)a$ and government transfers for low-income households are denoted by $TR(y) = t_r \mathbbm {1}_{TR}(y)$, where $\mathbbm {1}_{TR}(y)=1$ if $y<\bar{t_r}$ and zero otherwise, as explained above. While working, backpack asset $b'$ accumulate in the worker’s individual BP account, according to (17).

Equation (15) reads in the following way: the first term account the utility flow from consumption and labor. In the discounted continuation value, the expectation operator accounts for survival risk, all possible continuation histories of the realization of the stochastic component $z'\in {\mathcal {Z}}$, and two distinct labor market outcomes: With probability $1-\sigma _j$, the worker keeps the job in the next period (and therefore is not eligible to claim unemployment benefits), with value denoted *J* that depends on next period’s private and backpack assets, respectively $a'$ and $b'$, and the new realization of idiosyncratic productivity $z'$; alternatively, with probability $\sigma _j$, the job is destroyed and the worker starts next period without a job, with value *U*. This value depends on the number of periods after an involuntary job separation (relevant to determine eligibility for unemployment benefits), *d*. In the first period after a layoff, $d=0$. $z'$ follows the Markov chain described in (1).

Workers can start the period without a job. A job searcher who faced a job separation shock and has yet to start a new job has access to his backpack savings and, depending on how long he has been without working, may be eligible to receive unemployment benefits. He solves a consumption-savings problem, a job search problem, and a portfolio problem for the allocation of his private and backpack savings. At the beginning of the period, the individual state vector is given by private asset holdings *a*, backpack savings *b*, stochastic productivity *z*, and layoff duration *d*. Given the current state, the agent chooses consumption, future asset holdings and the search effort $e \in \{0,1\}$ according to:

19 $$\begin{aligned} U = \max _{c,a',b',e} \Bigg \{&u(c) - \gamma e + \beta {\mathbb {E}} \Big [ e \Big (\lambda ^{\mathbf{u }}_j J + (1-\lambda ^{\mathbf{u }}_j) U\Big ) + (1-e) \Big (\lambda ^{\mathbf{n }}_j J + (1-\lambda ^{\mathbf{n }}_j) N\Big ) \Big ] \Bigg \} \end{aligned}$$

subject to

20 $$\begin{aligned} (1+\tau _c) c + a' + b'(e) + \tau _y {\hat{y}} \le (1+r(1-\tau _k))(a + b) + UB(d,e) + TR(y),\qquad \end{aligned}$$

and

21 $$\begin{aligned} a', b'(e)&\ge 0. \end{aligned}$$

The first term in Eq. (19) inside the curly brackets is the flow utility from consumption and the utility cost of search, $\gamma e$. The expected continuation value takes into account the survival probability and the evolution of the stochastic productivity component, *z*. Higher search effort ($e=1$) translates into higher probability of finding a job: $\lambda ^{\mathbf{u }}_j>\lambda ^{\mathbf{n }}_j$. The trade-off in the job search problem is inside the expectation operator: With high search effort in the current period, with utility cost $\gamma$, the agent finds a job next period with probability $\lambda ^{\mathbf{u }}_j$; with low search effort ($e=0$), a job arrives with lower probability $\lambda ^{\mathbf{n }}_j$. If the worker finds a job, he decides in the beginning of next period whether to work or not at that job, with an option value *J* which depends on beginning of period assets and labor productivity. If search is not successful the worker continues unemployed next period with probability $(1-\lambda ^{\mathbf{u }}_j)$, with value *U* which depends on assets, productivity and unemployment duration $d'=d+1$. If the unemployed worker decides not to search ($e=0$) and does not find a job, he becomes non-eligible for unemployment insurance benefits and may again search for a job next period, with associated value *N*.

Equation (20) is the budget constraint. Total income is used to finance consumption expenditures, next period assets and income taxes, with the income tax base given by ${\hat{y}} = r(1-\tau _k)a$. The right-hand side is the sum of beginning of period private and backpack assets, plus after-tax return, unemployment benefits *UB*(*d*, *e*) and government transfers for low-income households, *TR*(*y*). The laid-off worker may be entitled to unemployment benefits: $UB(d,e) = u_b \mathbbm {1}_{UB}(d,e)$, with $\mathbbm {1}_{UB}(d,e)=1$ indicating eligibility for unemployment benefits. Formally:

22 $$\begin{aligned} \mathbbm {1}_{UB}(d,e) = {\left\{ \begin{array}{ll} 1 &{}\text {if } e = 1 \text { and } d \le {\bar{d}}, \\ 0 &{}\text {otherwise}. \end{array}\right. } \end{aligned}$$

The state variable *d* evolves deterministically according to $d'=d+1$ if the worker continues unemployed in the following period, and $d=0$ in the period immediately after a separation shock. We make two important simplifying assumptions here. The search effort is dichotumous: Either can the agent actively search for a job ($e=1$), or he does not search ($e=0$). Additionally, the possibility of using backpack assets while unemployed and searching for a job is represented by $b'(e)$ in the constraint (20): The laid-off worker can use his backpack savings to finance present (or future) consumption if he searches for a new job, but cannot increase backpack holdings other than through wage contributions (i.e., while working). Formally:

23 $$\begin{aligned} b'(e) {\left\{ \begin{array}{ll} \le &{} \tau _b y + (1+r(1-\tau _k))b,\text {if } e = 1 , \\ =&{} \tau _b y + (1+r(1-\tau _k))b,\text {if } e = 0. \end{array}\right. } \end{aligned}$$

The fact that unemployed eligible ($e=1$) workers can use some or all of accumulated BP savings is reflected in the $\le$ sign, above. There is a non-borrowing constraint given by (21).

Finally, an individual can start the period without a job because he has decided not to work or not to search in previous periods, not having found a new job yet. In this scenario, he solves the following problem:

24 $$\begin{aligned} N = \max _{c,a',e} \Bigg \{&u(c) - \gamma e + \beta {\mathbb {E}} \Big [ e \Big (\lambda ^{\mathbf{u }}_j J + (1-\lambda ^{\mathbf{u }}_j) N\Big ) + (1-e) \Big (\lambda ^{\mathbf{n }}_j J + (1-\lambda ^{\mathbf{n }}_j) N\Big ) \Big ] \Bigg \}, \end{aligned}$$

subject to

25 $$\begin{aligned} (1+\tau _c) c + a' + \tau _y {\hat{y}} \le (1+r(1-\tau _k))a + TR(y), \end{aligned}$$

and

26 $$\begin{aligned} a'&\ge 0, \end{aligned}$$

27 $$\begin{aligned} b'&= (1+r(1-\tau _k))b . \end{aligned}$$

As above, ${\hat{y}} = r(1-\tau _k)a$. The decision problem is similar to (19). But in this case, the unemployed worker is not eligible for unemployment benefits, and he also cannot use backpack assets. Accordingly, the evolution of BP assets is given by (27).

Retired individuals are not in the labor market and have no endowment of efficiency units of labor. They finance consumption with past private savings, backpack annuity payments, and PAYG pension payments. The problem is a standard consumption-savings decision, with survival risk and a certain maximum attainable age, assumed to be $j=100$. At age $j=99$, the continuation value is zero because the agent exits the economy next period with probability one. During retirement, the retired household solves a standard consumption-savings problem taking into account survival probabilities and total income:

28 $$\begin{aligned} V (a) = \max _{c,a'} \Bigg \{ u(c) + \beta {\mathbb {E}} \Big [ V (a') \Big ] \Bigg \} , \end{aligned}$$

subject to

29 $$\begin{aligned} (1+\tau _c) c + a' + \tau _y {\hat{y}} \le (1+r(1-\tau _k))a + p^S_h + p^B(b) + TR(y). \end{aligned}$$

Pension payments and backpack annuities are part of the income side of the budget constraint. In this case, ${\hat{y}} = r(1-\tau _k)a + p^S_h + p^B(b)$. After retirement, labor market productivity is always zero, and hence, expectations take into account only the survival risk.

To close the description of the household’s problem, we define the job acceptance and retirement decisions. These jointly pin down the value of having a job offer at the beginning of a period. For a household older or at the minimum retirement age (as defined by the PAYG pension system rules), $j\ge R_0$:

30 $$\begin{aligned} J&= \max \Big \{ V, \max \{ W, N \}\Big \}. \end{aligned}$$

The outermost $\max$ operator represents the retirement decision, while the inner operator is the job acceptance decision. Younger households, $j<R_0$, make only the job acceptance decision.

### Stationary equilibrium

The formal definition of a stationary equilibrium in the open economy, as well as in the closed economy, is postponed to Appendix G.

## Calibration

In order to calibrate the model parameters using Spanish data, we need to modify the environment described in Sect. 2. These modifications are, however, restricted to the elimination of the BP system, and therefore, the decision problem facing households, described above, is almost unchanged. Specifically, in this economy there is no Backpack fund, backpack assets (and contributions) are zero and claims on future consumption take only two forms: private savings and government retirement pensions. Henceforth, we use the following designation:

*Baseline economy*. The status quo economy, calibrated to the Spanish data in 2018, which includes a pay-as-you-go retirement pension system (see Appendix 1 for details about the PAYG system). There is no Backpack system: $\tau _b=0$.

To calibrate our model economy we choose a calibration target country, Spain in this article, and a calibration target year: 2018. We then choose the initial conditions and the parameter values that allow our model economy to replicate as closely as possible selected macroeconomic aggregates and ratios, distributional statistics, and the institutional details of our chosen country in the target year. More specifically, to characterize our model economy fully, we must choose the values of 42 parameters. To choose these parameter values, we need 42 equations or calibration targets. We describe the calibration process in all detail, including the data sources, in Appendix A (Table 1).

**Fig. 1:**
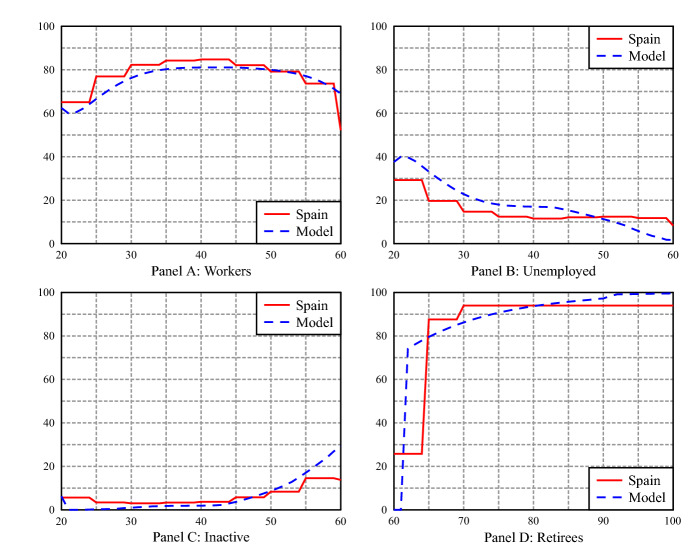
Labor market stocks by age in the data and in the model. Data source is the survey is Encuesta de Población Activa

An important assumption is that in our benchmark economy we treat Spain as a small open economy. This means that the interest rate is constant, and therefore, for the competitive representative firm the capital–labor ratio and the wage rate are given and constant. In Sect. 5, we analyze Spain as a closed economy and, therefore, the general equilibrium effects of introducing the backpack. Following our open economy benchmark^7^, the next section presents the most relevant calibration targets and model statistics, as well as the government’s budget components (Fig. 1).

### Baseline economy

The following tables summarize our calibration. The values shown in boldface are our data targets.

**Table 1:** Macroeconomic aggregates and ratios in Spain and in the model, in 2018

	$K/Y^{*}$	$C/Y^{*}$	$I^{a}/Y^{*}$	*h*
Spain	2.94	50.70	26.95	34.59
Model	3.06	41.76	34.90	33.11

Variable $Y^{*}$ denotes GDP at market prices.

$$I^{a}$$ denotes investment.

*h* denotes average share of disposable time allocated to market work.

All columns except the first are in percentage.

Data source: Fundación BBVA and Spanish National Institute of Statistics (INE)

**Table 2:** Macroeconomic aggregates and ratios in Spain and in the model, in 2018

	$P/Y^{*}$	$U/Y^{*}$	$T_{r}/Y^{*}$	*GW*	*W*	*I*
Spain	**10.47**	**1.32**	**0.83**	**0.67**	**59.59**	**5.16**
Model	10.54	1.15	0.88	0.68	58.50	4.93

$Y^{*}$ denotes GDP at market prices.

$U/Y^{*}$ is unemployment benefits as a share of output.

*GW* is the Gini Index of wealth.

*W* is the share of workers in the Spanish population with 20+ years old.

*I* is the share of inactive in the Spanish population with 20+ years old.

Data source: Spanish National Institute of Statistics (INE), Spanish Social Security, Cañón et al. (2016); Anghel et al. (2018)

The values shown in boldface are the data targets for our calibration

**Table 3:** Government budget in Spain and in the model, in 2018 ($\%$ of output at market prices)

	Public Expenditure				Public Revenues				
	*G*	$T_{r}$	*P*	*U*	$T_{c}$	$T_{k}$	$T_{y}$	$T_{p}$	*E*
Spain	**17.40**	**0.83**	**10.47**	**1.32**	9.07	**2.24**	**7.05**	9.47	**0.20**
Model	17.40	0.88	10.54	1.15	8.68	2.33	7.05	11.67	0.20

*G*: government consumption, $T_r$: welfare transfers, *P*: pension payments, *U*: unemployment benefits expenditures; $T_c$: consumption tax collections, $T_k$: capital income taxes, $T_y$: household income tax revenue, $T_p$: payroll tax revenue, *E*: accidental bequests revenue.

Data source: Spanish Social Security (Resumen de Ejecución del Prespuesto) and Spanish National Institute of Statistics (Cuentas Nacionales)

The values shown in boldface are the data targets for our calibration

The model is able to capture the main output ratios in the calibrated year, as shown in Table 2. As shown in Table 3, we target government expenditures and revenue ratios in order to determine the simplified tax system in the model. The payroll tax rate finances pension and unemployment benefit expenditures. Capital income and household income tax rates are chosen to collect 2.24% and 7.05% of GDP (at market prices), as it is the case in Spain in 2018. Finally, the consumption tax rate clears the government budget. Some Spanish regions feature a proportional tax on bequests. We use the aggregate revenue of this tax in 2018 as the data point for *E* (0.20% of output). In the model, aggregate accidental bequests as a fraction of output are significantly higher (2.63). In the results shown below, we assume that the portion of the accidental bequests that is not taxed by the government is wasted (thrown to the sea).

The tax rates implied by the calibration are shown in Table 4.

**Table 4:** Policy Parameters in the model economy, in 2018

	Tax rates (%)			
	$\tau _c$	$\tau _y$	$\tau _k$	$\tau _p$
Model	26.2	14.2	25.0	26.0

$\tau _c$: consumption tax rate, $\tau _y$: household income tax rate, $\tau _k$: capital income tax rate, $\tau _p$: payroll tax

The model also does a good job at replicating the aggregate labor market stocks (share of workers and inactive targeted in the calibration), and the age distribution of workers, unemployed, inactive, and retirees—which is not part of the calibration targets.

**Table 5:** Labor Market Shares in 2018 (% of population)

	W	U	I	R
Spain	**59.59**	10.72	**5.16**	24.51
Model	58.50	11.92	4.93	24.65

*W*: workers, *U*: unemployed, *I*: inactive, *R*: retirees.

Data source: Encuesta de Población Activa (INE)

The values shown in boldface are the data targets for our calibration

Our model features labor market frictions and a detailed description of government tax and transfer systems and is able to capture the inequality in after-tax earnings, income and net wealth in the Spanish economy, as summarized in Table 6.

**Table 6:** Inequality in Spain and in the model in 2018$^{*}$

	GE	GI	GW
Spain	0.34	0.33	**0.67**
Model	0.34	0.36	0.68

*GE*: Gini Index of net earnings, *GI*: Gini Index of net income, *GWI*: Gini Index of net wealth.

$^{*}$The source for the Spanish data of earnings and income are the Spanish National Institute of Statistics (INE) and the OECD. The source for the Spanish data of wealth is BDE (2018)

## Spain, as an open economy, with the backpack

**Fig. 2:**
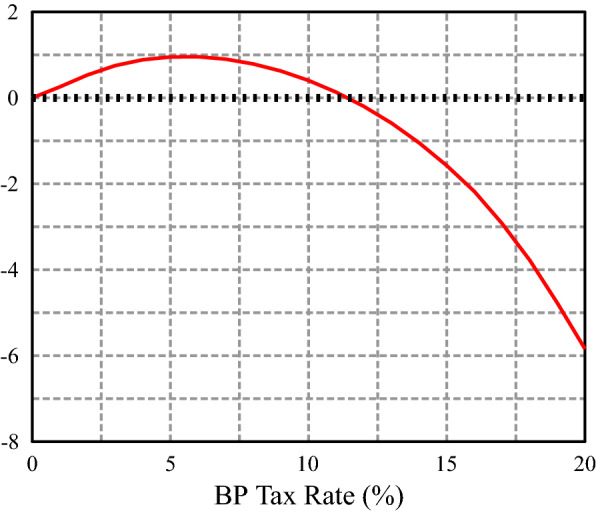
Consumption equivalent variation (CEV) in average lifetime utility for newborn agents

In this section, we show the effect of introducing the backpack in Spain while maintaining the existing unemployment insurance (UI) and pension systems. We first compare different steady states indexed by the contribution rate, and calculate the difference in average social welfare relative to the baseline economy (without backpack system). $\tau _b=6.0\%$ emerges as the welfare maximizing backpack rate (Fig. 2). More precisely, we take as welfare criteria the average lifetime utility of a 20 years old agent about to enter the economy. The average is calculated using the cross-sectional probability distribution over the initial states (education, productivity, employment status). We then analyze in more detail the backpack economy.

The introduction of the backpack with its corresponding contribution rate, while fully funded, does not imply that the government budget is satisfied in the new steady state since, for example, revenue from income taxation may be lower (agents’ decisions, the income tax base, change). The assumption we follow is to fix government consumption *G* at the baseline level, and let $\tau _c$ adjust to clear the budget.^8^ Similarly, we allow the payroll tax to adjust to changes on unemployment benefits or pensions, according to (11), so that the Social Security budget is balanced. Income and capital tax rates remain unchanged.

In general, individual decisions and aggregate variables are affected by the introduction of the backpack. At the individual level, it affects decisions on savings, since accumulating BP assets is a form of forced savings, subject to dis-investment restrictions, with two advantages: It reduces the need for precautionary private savings and, in contrast to private savings, is not subject to household income taxation. It also affects consumption, since, on the one hand, it helps to smooth consumption after a job loss, when jobless individuals can withdraw from their backpack, while on the other hand the retirement annuity implies an increase in lifetime consumption. Additionally, it affects labor supply, since consumption and saving plans are affected, direct labor income taxes are likely to be lower, while effective labor taxes may be higher, and importantly, there is an incentive to postpone retirement and further accumulate BP assets.^9^ The introduction of the BP has an effect on individual decisions. These in turn, depending on the overall distribution of the population (age, education and productivity profiles), have an effect on aggregate savings, consumption and employment. These effects also translate into welfare differences between the benchmark and the economy with a backpack. We evaluate social welfare across the different economies by calculating the implied consumption equivalent variation (CEV) in the expected lifetime utility of individuals entering the economy at age 20, relative to the benchmark model that is calibrated to the Spanish economy in 2018. We calculate the welfare changes for specific groups in the population, depending on education, labor market productivity, and labor market status.

We now quantify the effects of introducing the BP in the (open) Spanish economy. Table 7 shows the aggregate effects. While private asset holdings go down, the backpack savings imply that, at the new stationary equilibrium, the total assets are 31% larger. Aggregate consumption increases by 3.1%, while the effect on total hours work is small—an increase of 0.8%. Note that, as an open economy, interest rate and wages—and, therefore, the capital–labor ratio—are externally given. Therefore, the current account does not need to be balanced at the steady state; in fact, it is positive: its net value over GDP, $(S-K)/Y$, is 6.42$\%$ in the baseline economy and, slightly lower, 5.70$\%$ in the economy with the BP.

**Table 7:** Aggregates and output ratios in the baseline economy and in the economy with the BP system (BP Economy)

	Values			Y Ratios$^*$		
	Baseline	BP Economy	$\%\Delta$	Baseline	BP Economy	$\Delta p.p.^{a}$
Y	2.1595	2.1757	0.75	100.00	100.00	0.00
A	3.9348	5.1631	31.21	182.20	237.30	55.10
L	0.6912	0.6964	0.75	32.00	32.00	0.00
H	0.1936	0.1949	0.67	8.96	8.96	0.0
C	0.7822	0.8068	3.14	36.22	37.08	0.86
G	0.4114	0.4114	0.00	19.05	18.90	–0.15
I	0.8258	0.8316	0.78	38.24	38.24	0.00
w	1.6102	1.6102	0.00			
r (%)	3.0476	3.0476	0.00			

The BP Economy is the reformed economy with BP system. *Y*: output at factor cost, *A*: total assets, *L*: labor in efficiency units, *H* hours of labor supply, *C*: aggregate private consumption plus consumption tax collections, *G*: government consumption, *I*: investment, *w*: wage rate, *r*: interest rate.

$^{a}:$ Difference in percentage points.

${*}:$ As a share of output at factor cost

Table 8 shows the differences in taxes and government revenues. The income tax exemption on backpack asset income results in a decline of household income tax collection, which is compensated by small increases of revenues from capital and consumption taxation, the latter due to an increase of consumption which overall allows for a decrease of the consumption tax. Nevertheless, due to the introduced of an additional tax wedge, the final result is a 9.8% increase of the effective labor tax, $\tau _e$.

**Table 8:** Taxes and tax revenues in the baseline economy and in the backpack economy)

	Tax Rates			Tax Revenues			Revenue Y Ratios $(\%)^{*}$		
	Baseline	BP Economy	$\%\Delta$	Baseline	BP Economy	$\%\Delta$	Baseline	BP Economy	$\Delta p.p^{a}$
$\tau _c$	0.2624	0.2567	-2.18	0.2052	0.2071	0.93	9.50	9.51	0.01
$\tau _y$	0.1418	0.1417	0.00	0.1667	0.1643	-1.44	7.71	7.55	-0.16
$\tau _k$	0.2500	0.2500	0.00	0.0552	0.0556	0.72	2.55	2.55	0.00
$\tau _p$	0.2600	0.2619	0.73	0.2761	0.2807	1.66	12.78	12.90	0.12
$\tau _b$	-	0.0600	-	-	0.0672	-	-	3.08	-
$\tau _e$	0.4986	0.5434	9.78						

$\tau _c$: consumption tax rate, $\tau _y$: household income tax rate, $\tau _k$: capital income tax rate, $\tau _p$: payroll tax, $\tau _b$: backpack contribution tax, $\tau _e$: effective labor tax.

$^{a}:$ Difference in percentage points.

$^{*}:$ As a share of output at factor cost

In Table 9, we show the percentage of the population by labor status, and our findings show that, in spite of the increase of the effective labor tax, the backpack system increases labor market participation, with a small increase in employment. In particular, the percentage of inactive people decreases by more than 1.5 percentage points. Therefore, there is an equivalent increase in the active population arriving to the first retirement age, and even if the percentage of retirees is almost the same, retirement is being delayed in the BP economy by 0.3 years on average.

**Table 9:** Labor market shares (% of population)

	W	U	I	R
Baseline	58.50	11.92	4.93	24.65
BP economy	58.99	12.92	3.40	24.69

*W*: workers, *U*: unemployed, *I*: inactive, *R*: retirees. All shares correspond to people aged 20+

**Fig. 3:**
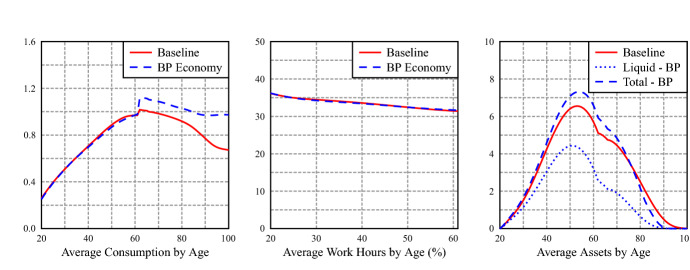
Average profiles of consumption, work hours and liquid assets by age

**Fig. 4:**
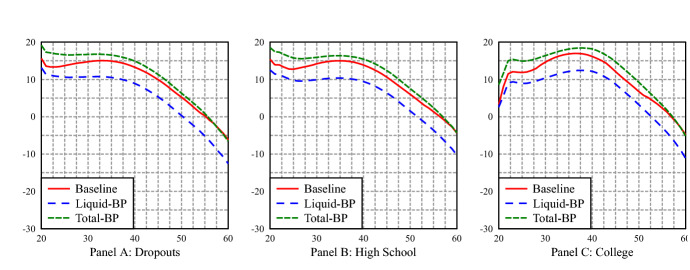
Saving rate relative to average gross earnings for workers by age

A closer look at average age profiles of consumption, hours of work and asset holdings, in Fig. 3, shows a slight decrease on average hours work, an important substitution from private liquid to BP assets, with an increase in total assets^10^. Interestingly, it also shows that the increase in consumption is mostly due to a substantial increase in consumption after retirement. The BP avoids, thanks to the annuity payment, the sharp decrease in consumption that occurs in the baseline economy. In the latter, pensions provide a stable source of income, but private assets are depleted in retirement (there is no bequest motive in our model), which translates into a decrease in consumption. Figure 4 shows that total savings (include BP contributions) are slightly higher in years close to retirement in the baseline economy: households deplete the stock of liquid asset at a lower rate, due to lower retirement income. Nevertheless, the sharp increase of consumption upon retirement reflect a distortion to a smooth life consumption pattern. There is a jump in the average profile of consumption at the first retirement age. The small jump in consumption at retirement in the PAYG economy is mainly driven by the jump in consumption of inactive households, while in the reformed PAYG+BP economy, the jump in consumption is because all groups significantly increase their consumption when moving from 61 to 62 years old. Appendix E contains a detailed analysis of consumption in both economies for different age and labor market groups.

In comparing status quo and reformed economies, welfare and inequality measures are most relevant and we close the section with these results. As already mentioned, our welfare measures are in terms of lifetime consumption improvement, expected by a new entrant in the economy (age 20). Therefore, the back-loaded increase of consumption at retirement age in the BP economy is properly discounted by the young workers. Taking into account the distribution by types (education, initial productivity and labor status), the overall social welfare gains are significant: 0.95%. The breakdown of the welfare gains across different groups is reported in Tables 10 and 11. We find that, in general, welfare gains decrease with the education. This is mainly because more educated workers start to save at older ages in the baseline economy; consequently, introducing a forced saving of 6$\%$ of earnings from the start of their productive careers, hurts them the most. In fact, the only group which is harmed by the introduction of the BP is the group of the low productive college educated. Table 11 shows how unemployed and inactive are better off in the economy with a backpack system, mainly because the unemployment insurance provided by the backpack system and the better alternatives to inactivity that the BP economy offers. The welfare gains for the retirees are counted from age 62 (the earliest retirement age in the baseline economy), and as their consumption profile of Fig. 3 reveals, they are the group who benefits the most from the introduction of the backpack.

**Table 10:** Consumption equivalent variation (%) in average lifetime utility in the economy with a backpack system (BP), relative to the baseline economy: workers

Education	Labor Productivity		
	Low	Medium	High
Dropouts	0.86	1.18	2.10
High school	0.74	1.05	1.58
College	-0.08	0.79	1.12

**Table 11:** Average consumption equivalent variation (%) in the economy with a backpack system (BP), relative to the baseline economy: unemployed, inactive, and retirees. For retirees, we use average remaining lifetime utility at the first retirement age

Labor status	Education		
	Dropouts	High school	College
Unemployed	1.09	1.03	0.78
Inactive	1.09	1.04	0.79
Retirees	4.08	3.98	3.61

Regarding inequality, moving from the baseline to the BP economy increases wealth inequality since low earners (those who receive the lowest stochastic productivity shock) reduce their holdings of liquid assets proportionately more than those medium and high earners and the latter—in particular, the top 5$^{\mathrm{{th}}}$ quintile—experience the highest accumulation of assets. Specifically, the Gini of Wealth increases from 0.68 to 0.76 (see Table 18). There is no significant variation in earnings inequality, while income inequality slightly increases from 0.36 to 0.37, mainly because of the increase in capital income inequality, with the 3$^{\mathrm{{rd}}}$ and 4$^{\mathrm{{th}}}$ quintile being the beneficiaries in relative terms.

**Table 12:** Distributions of earnings, income, and $\hbox {wealth}^{*}$

		Bottom	Quintiles					Top
	Gini	10	1st	2nd	3rd	4th	5th	10
The Earnings Distributions $(\%)$								
Baseline	0.34	3.6	8.4	11.4	15.6	23.5	41.1	26.3
BP Economy	0.34	3.6	8.4	11.4	15.7	23.5	41.1	26.3
The Income Distributions $(\%)$								
Baseline	0.36	1.9	5.9	12.9	15.0	22.1	44.0	27.8
BP Economy	0.37	1.9	5.7	12.1	16.8	23.3	42.1	26.6
The Wealth Distributions $(\%)$								
Baseline	0.68	0.0	0.0	2.4	7.8	21.4	68.4	47.4
BP Economy	0.76	0.0	0.0	0.3	3.6	19.1	76.9	54.8

## The effect of the backpack in Spain, as a closed economy.

In this section, we consider Spain as a closed economy. The general equilibrium effects exacerbate the differences between the BP and the baseline economy. We first need a new calibration for the baseline as a closed economy (see Appendix C for details). We proceed as in Sect. 4. Figure 6 shows how the welfare maximizing backpack rate is much higher: a 18% BP rate (three times the open economy rate).

**Fig. 5:**
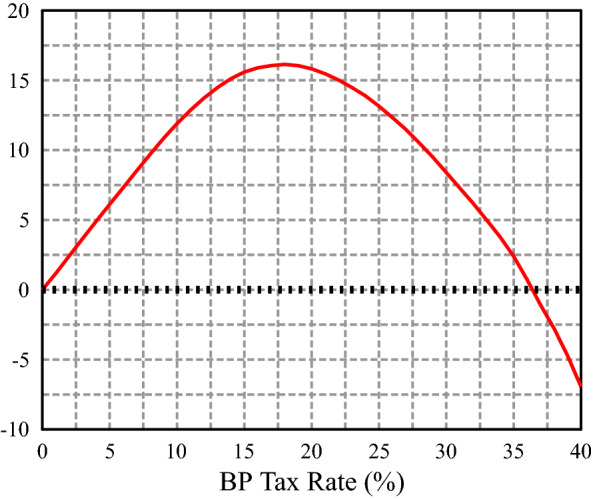
Consumption equivalent variation (CEV) in average lifetime utility for newborn agents

Table 22 shows first how in the stationary equilibrium of the baseline closed economy, the interest rate is higher and wages lower than in the corresponding equilibrium of open economy described in Table 7, and as a result, other aggregates are smaller (except for consumption). Second, and more importantly, it shows how the impact of introducing a backpack is larger; in particular, the interest rate is lower and the reference wage is higher. The economy is more capitalized, and with the exception of hours worked, all the aggregates are larger than in the (closed) economy without the BP.

**Table 13:** Aggregates and output ratios in the baseline (closed) economy and in the economy with BP system

	Values			Y $\hbox {Ratios}^{*}$		
	Baseline	BP	$\%\Delta$	Baseline	BP	$\Delta p.p.^{a}$
Y	2.0419	2.6657	30.54	100.00	100.00	0.00
K	6.5850	11.4524	73.91	322.49	429.62	107.13
L	0.6785	0.6774	$-$0.17	33.22	25.41	$-$7.81
H	0.1911	0.1907	$-$0.21	9.35	7.15	$-$2.20
C	0.9459	1.1080	17.13	46.32	41.56	$-$4.76
G	0.3891	0.3891	0.00	19.05	14.59	$-$4.46
I	0.7068	1.1657	64.92	34.61	43.72	9.11
w	1.5495	2.0297	30.99			
r ($\%$)	6.4441	2.6935	$-$58.21			

The BP Economy is the reformed economy with a BP system. *Y*: output at factor cost, *K*: capital stock, *L*: labor in efficiency units, *H* hours of labor supply, *C*: aggregate private consumption plus consumption tax collections, *G*: government consumption, *I*: investment, *w*: wage rate, *r*: interest rate.

$^{a}:$ Difference in percentage points.

${*}:$ As a share of output at factor cost

The aggregate changes due to the introduction of the backpack have an important impact on tax revenues. As Table 14 shows, with a 18% backpack contribution tax, the effective labor tax is larger than in the baseline economy (and larger than in the open economy with a BP). With a lower interest rate, revenues from capital taxation are 27% lower, which is compensated with higher revenues from income and consumption taxes; the latter thanks to an increase of consumption, which allows for a decrease of the consumption tax.

**Table 14:** Policy parameters and tax revenue ratios in the baseline (closed) economy and in the economy with the BP system (BP economy)

	Tax rates			Tax revenues			Revenue Y ratios $(\%)^{*}$		
	Baseline	BP economy	$\%\Delta$	Baseline	BP economy	$\%\Delta$	Baseline	BP economy	$\Delta p.p^{a}$
$\tau _c$	0.2059	0.1907	$-$7.39	0.1947	0.2113	8.52	9.53	7.92	$-$1.61
$\tau _y$	0.1126	0.1126	0.00	0.1577	0.1643	4.18	7.72	6.16	$-$1.56
$\tau _k$	0.1184	0.1184	0.00	0.0502	0.0365	$-$27.30	2.45	1.36	$-$1.09
$\tau _p$	0.2587	0.2740	5.91	0.2604	0.3658	40.47	12.75	13.72	0.97
$\tau _b$	–	0.1800	–	–	0.2475	-	-	9.28	-
$\tau _e$	0.4546	0.5954	30.97						

$\tau _c$: consumption tax rate, $\tau _y$: household income tax rate, $\tau _k$: capital income tax rate, $\tau _p$: payroll tax, $\tau _b$: backpack contribution tax, $\tau _e$: effective labor tax.

$^{a}:$ Difference in percentage points.

$^{*}:$ As a share of output at factor cost

As Table 15 shows, active population increases by 3%, in spite of the increase of the effective labor tax. The latter in part due to an increase of the payroll tax to finance the increase of unemployment benefits and pensions corresponding to the larger fractions of unemployed and retirees. In sum, the labor allocation is more efficient with a decrease of the inactive population by more than 3 percent points.

**Table 15:** Labor market shares (% of population)

	W	U	I	R
Baseline	58.39	11.76	5.14	24.69
BP economy	58.42	13.66	2.20	25.71

*W*: workers, *U*: unemployed, *I*: inactive, *R*: retirees

**Fig. 6:**
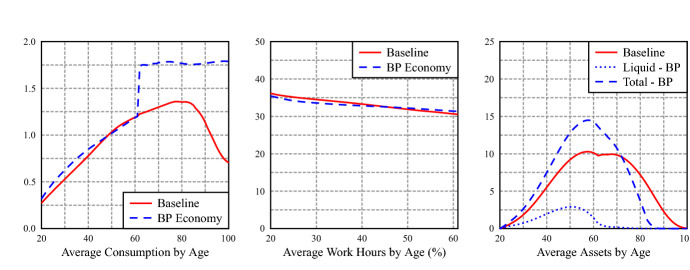
Average profiles of consumption, work hours and liquid assets by age

Figure 6 shows how, in comparison with the open economy, there is a larger substitution of private liquid assets for backpack assets and, with a jump of consumption upon retirement also widely exacerbated. Consumption jumps at retirement due to the increase in disposable income for all education groups, which is composed of PAYG retirement pensions and BP annuity, and to the borrowing limit before retirement that prevents households from borrowing against this income and increase consumption prior to retirement.^11^

The comparison of the welfare gains in the closed economy of Tables 16 and 17 with those of the open economy (Tables 10 and 11) shows that welfare gains are uniformly higher for all groups of the young generation. In the closed, everyone gains from having a BP, and in particular, dropouts gain the most.

**Table 16:** Consumption equivalent variation (%) in average lifetime utility in the economy with a backpack system (BP), relative to the baseline (closed) economy: workers

Education	Labor productivity		
	Low	Medium	High
Dropouts	15.74	11.47	4.34
High School	16.40	13.55	6.22
College	15.55	16.68	10.79

**Table 17:** Consumption equivalent variation (%) in the economy with a backpack system (BP), relative to the baseline (closed) economy: unemployed, inactive, and retirees

Labor Status	Education		
	Dropouts	High School	College
Unemployed	17.39	18.63	20.11
Inactive	17.94	19.14	20.29
Retirees	12.43	13.00	13.21

**Table 18:** Distributions of earnings, income, and $\hbox {wealth}^{*}$

		Bottom	Quintiles					Top
	Gini	10	1st	2nd	3rd	4th	5th	10
The Earnings Distributions $(\%)$								
Baseline	0.33	3.7	8.5	11.4	15.8	23.5	40.9	26.0
BP economy	0.33	3.7	8.5	11.4	15.5	23.4	41.1	26.3
The Income Distributions $(\%)$								
Baseline	0.37	2.1	5.9	11.8	15.3	22.8	44.3	28.0
BP economy	0.37	2.0	5.6	10.2	16.5	24.7	43.1	26.3
The Wealth Distributions $(\%)$								
Baseline	0.60	0.0	0.8	4.8	10.4	22.7	61.2	40.7
BP economy	0.84	0.0	0.0	0.0	0.1	10.0	89.8	67.6

## The role of BP annuity payments

**Fig. 7:**
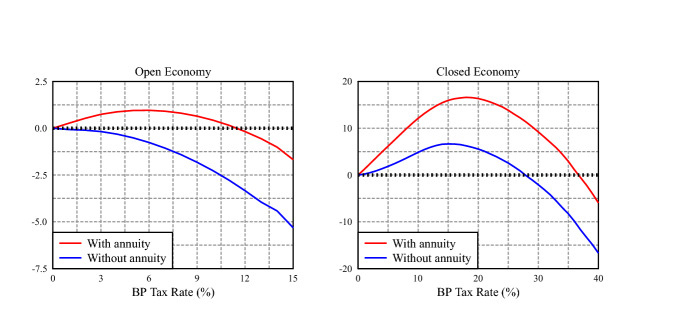
Consumption equivalent variation (CEV) in average lifetime utility for newborn agents

Our backpack contract adds to the Austrian backpack the option to convert the backpack savings, upon retirement, into an actuarially fair annuity, providing insurance akin to the retirement insurance of a PAYG pension. Hence, the paper’s motivation of studying this policy and contrasting it to existing UI and PAYG pension systems. In this section we show that this feature is an important component of the welfare gains described in Sects. 4 and 5. As the left panel of Fig. 7 shows, if in the open economy of Sect. 4 we remove the annuity option—an option that all agents were choosing—and, therefore, backpack assets become just part of their liquid private assets, the welfare maximizing backpack rate is $\tau _b = 0.0\%$; that is, there are no welfare gains from introducing the Austrian backpack without the annuity option. In contrast, as the right panel of Fig. 7 shows, introducing the Austrian backpack without the annuity in the closed economy results in welfare gains of 6.6%, with a welfare maximizing backpack rate of $\tau _b = 15.0\%$, instead of $\tau _b = 18.0\%$ when (Section 5) the annuity option is available.

**Fig. 8:**
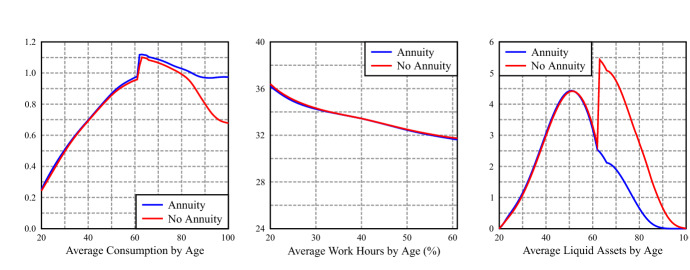
Average profiles of consumption, work hours and liquid assets by age: BP economies with and without annuities

Figure 8 shows the average age profiles of consumption, hours of market work and asset holdings of the open economy with a backpack rate of $\tau _b = 6.0\%$ without annuity, in comparison with the profiles of Fig. 3 in Sect. 4. The jump of consumption at the first retirement age is the same, but consumption decays with retirement, as it does in the benchmark PAYG economy (Fig. 3), while liquid assets jump, given the non-annuitized backpack assets inflow. Conditional on having a backpack system, the annuity option dominates in welfare terms, but as we have seen, it is this welfare gain that justifies the introduction of the backpack in the open economy, while in a closed economy it only plays a complimentary role in the overall increase in welfare.

These results raise an obvious issue: should the annuity option be available for all liquid assets? It could, and should, since it is a dominant contract when agents want to insure their longevity risk. Nevertheless, private annuity contracts exists but this is a very thin market, raising concerns regarding its ‘actuarially fairness,’ which is easier to achieve in a thick—possibly regulated—insurance market. The introduction of the BP system is an occasion to establish a competitive and actuarially fair annuity market. The role of supervision and competition policy is better anchored when it is a complement to a public policy generating a broad base for this market. It is for this reason that we do not include this option in our analysis and emphasize the social value of developing the retirement annuity market.

## Conclusions

We have studied the effect of the introduction of the Austrian backpack contract (BP) as complement to other existing social policies—in particular, unemployment insurance and a PAYG pension system—using the Spanish economy in 2018 as a benchmark for our calibration and experiments. By studying the BP in itself, and not as a part of a broader reform, we have been able to isolate its more salient features and effects. To this end, we have used a detailed overlapping generations model with heterogeneous agents making decisions on employment, savings and consumption, subject to idiosyncratic risk. We compare stationary equilibria without and with the BP. As a baseline, we consider Spain as an open economy. The basic element that defines the size of the BP is the payroll contribution tax. We choose the one that maximizes a Benthamite social welfare function of the generation entering into the labor market for the first time (20 years old). In our exercise, the optimal contribution rate is a 6% tax, resulting in small, but significant, aggregate effects. Nevertheless, behind them, there are multiple effects on individual decisions. All groups of agents, but one, benefit from the introduction of a 6% BP. With the BP, they can reduce their precautionary savings and substitute liquid private assets by income tax-free BP assets; smooth better their consumption during unemployment spells and during their retirement.

Even if the Spanish economy is part of the European Union, the European Financial Union is not as developed as to consider Spain a fully open economy. For this reason, we also analyze it as a closed economy. There are important general equilibrium effect of introducing the Austrian backpack: the economy capitalizes, with wages increasing and the interest rate decreasing. As a result, the welfare maximizing backpack rate is larger, 18%, as well as the corresponding welfare gains.

This comparison between the open and closed economy is also relevant when we analyze the role of the link between lifetime BP savings and annuity payments after retirement. It accounts for the average welfare gains of introducing the backpack system in the open economy, while it plays a complementary welfare improving role in the closed economy, for which, without the annuity option, the welfare maximizing backpack rate would be 15%. Therefore, we conclude that the improved Austrian backpack design is the appropriate one, since it also helps to sustain an annuity insurance market.

We unveil a special feature of how the BP contract interacts with PAYG pensions: for good and for bad, the BP contract backloads a substantial part of its benefits into the retirement age. For good, avoiding the drop of consumption in old age that characterizes the ‘only PAYG’  benchmark economy. For bad, because the *minimum retirement age*, together with the inability to borrow against future income (PAYG pension plus BP annuity), introduce a distortion into agents’ intertemporal consumption plans: upon retirement there is a jump in consumption when backpack assets become liquid^12^. In spite of this distortion, the economy is (slightly) more efficient with the BP, in terms of allocation of resources and productivity, and social welfare improves by 0.96%, with Spain as an open economy.

Finally, we also show (in Appendix) that, in fact, the BP is not a good substitute for the existing unemployment insurance system; the problem being the insufficient unemployment insurance coverage that the BP provides at the early stages of workers’ careers. In contrast, in a follow-up paper, Brogueira de Sousa et al. (2021), we show that the BP not only is an excellent replacement of the PAYG system, particularly in an aging society, but that it also dominates—in welfare and efficiency terms—other funded private or public systems. There, we also analyze the transitional reform of the pension system and its interaction with the Spanish aging transition in the first half of the twenty-first century.

## A. Calibration

### A.1 Initializing the steady state

In order to determine the steady state, first we choose as an initial distribution of households $\mu _0=\mu _{2018}$; that is, we take $\mu _{jh}$ at year 2018 directly from the Encuesta de Población Activa from the Spanish National Institute of Statistics. We also take from INE the conditional probabilities of surviving from age *j* to age $j+1$, $\psi _j$, at that same year. The labor market flow data used to calibrate the job finding and job destruction probabilities were provided by Lalé and Tarasonis (2017). The initial distribution of households imply an initial value for the capital stock. This value is $K_{2018}=7.2484$. Finally, the initial distribution of households and the initial survival probabilities determine the initial value of unintentional bequests, $E_{2018}$.

### A.2 Parameters

Once the initial conditions are specified, to characterize our model economy fully, we must choose the values of a total of 42 parameters. Of these 42 parameters, 5 describe the household preferences, 21 the process on the endowment of efficiency labor units, 3 the production technology, 4 the pension system rules, and 9 the remaining components of the government policy. The functional form for the utility function is $u(c,l)=\frac{c^{(1-\sigma )}}{1-\sigma }-\alpha \frac{l^{1+1/\varphi }}{1+1/\varphi }$. To choose the values of these 42 parameters we need 42 equations or calibration targets which we describe below.

### A.3 Equations

To determine the values of the 42 parameters that identify our model economy, we do the following. First, we determine the values of a group of 36 parameters directly using equations that involve either one parameter only, or one parameter and our guesses for (*Y*, *L*). To determine the values of the remaining 6 parameters we construct a system of 6 nonlinear equations. Most of these equations require that various statistics in our model economy replicate the values of the corresponding Spanish statistics in 2018. We describe the determination of both sets of parameters in the subsections below.

**Table 19:** Parameters determined solving single equations

	Parameter	Value
Parameters determined directly		
*Earnings Life Cycle*		
	$\xi _{1,1}$	0.9189
	$\xi _{1,2}$	0.8826
	$\xi _{1,3}$	0.5064
	$\xi _{2,1}$	0.0419
	$\xi _{2,2}$	0.0674
	$\xi _{2,3}$	0.1648
	$\xi _{3,1}$	0.0006
	$\xi _{3,2}$	0.0008
	$\xi _{3,3}$	0.0021
	*z*(1)	1.0000
	*z*(2)	2.3490
	*z*(3)	5.9042
	$z_{11}$	0.9821
	$z_{12}$	0.0177
	$z_{13}$	0.0001
	$z_{21}$	0.0291
	$z_{22}$	0.9709
	$z_{23}$	0.0000
	$z_{31}$	0.0001
	$z_{32}$	0.0003
	$z_{33}$	0.9995
*Preferences*		
Curvature	$\sigma$	2.0000
Labor elasticity	$\varphi$	0.1000
*Technology*		
Capital share	$\theta$	0.4846
Depreciation rate	$\delta$	0.1138
Interest rate	*r*	0.0304
*Public pension system*		
Number of years of contributions	$N_{b}$	21
First retirement age	$R_{0}$	62
Parameters determined by guesses for (*Y*, *L*)		
*Public pension system*		
Payroll tax rate	$\tau _{p}$	0.2597
*Government policy*		
Public debt	*D*	0.0000
Government consumption	*G*	0.3894
Accidental bequest	*E*	0.0047
Replacement rate	$b_{1}$	0.7500
Capital income tax rate	$\tau _{k}$	0.1188
Consumption tax rate	$\tau _{c}$	0.2064
Income tax rate	$\tau _{y}$	0.1128

#### A.3.1 Parameters determined solving single equations

*The life cycle profile of earnings.* We measure the deterministic component of the process on the endowment of efficiency labor units independently of the rest of the model. We estimate the values of the parameters of the three quadratic functions that we describe in Expression (31), using the age and educational distributions of hourly wages reported by the *Instituto Nacional de Estadística* (INE) in the *Encuesta de Estructura Salarial* (2010) for Spain.^13^ This procedure allows us to identify the values of 9 parameters directly.

31 $$\begin{aligned} \epsilon _{jh}=\xi _{1h}+\xi _{2h}j-\xi _{3h}j^{2} \end{aligned}$$

In order to determine the values for both the stochastic component of the process on the endowment of efficiency labor units and the conditional transition probabilities across them, we follow the procedure proposed by Castañeda et al. (2003)—this procedure gives us a total of 8 additional equations.

*The pension system* In 2018 in Spain, the payroll tax rate paid by households was 28.3 percent and it was levied only on the first 45,014 euros of annual gross labor income. Since we omit the tax cap, we impose that all gross earnings pay pension contributions. We also impose that payroll tax collections are used to finance both pension payments and unemployment benefits. This implies that the payroll tax rate in our model economy is 0.2609.

Our choice for the number of years used to compute the retirement pensions in our benchmark model economy is $N_{b}=21$. This is because in 2018 the Spanish *Régimen General de la Seguridad Social* took into account the last 21 years of contributions prior to retirement to compute the pension. Finally, our choice for the first retirement ages is $R_{0}=62$.

*Government policy* To specify the government policy, we must choose the values of government consumption, $G_{t}$, the level of public debt, $D_{t}$, the share of accidental bequest that is confiscated by the government, *E*, the replacement rate $b_{1}$, of the tax rate on capital income, $\tau _{k}$, of the tax rate on income, $\tau _{y}$, and of the tax rate on consumption, $\tau _{c}$.

We target the output shares of *G*, *E*, and $T_{y}$ so that they replicate the GDP shares of Government Consumption, Inheritance Taxes, and Individual Income taxes. According to the INE, in 2018, Government Consumption was 208,875 million euros, and the Inheritance Tax, and the Individual Income tax collected 2,687, and 93.247 million euros, respectively.^14^ Consequently, the ratios of these variables to GDP at market prices are 17.40, 0.20, and 7.05 percent.

For simplicity, we also assume that the initial level of public debt, as a share of GDP, is 0 percent. The rationale for this choice is because the government in our model economy has not the same number of tax instruments that the Spanish government, so we opt to discard this specific public expenditure. Otherwise, the consumption tax rate need to balance the government budget should increase to unrealistic levels. According to Cañón et al. (2016) the ratio of per capita public transfers to the threshold level was 75$\%$, so we set $b_{1}=0.75$. Regarding the capital income tax rate, and according to the OECD, in Spain in 2018, this number was 25$\%$, Consequently, we set $\tau _{k}=0.25$. Finally, the government budget is an additional equation that allows us to obtain residually the consumption tax rate.

*Preferences* Of the four parameters in the utility function, we choose the value of $\sigma$ and $\varphi$ directly. Specifically, we choose $\sigma = 2.0$ and $\varphi =0.1$.

*Technology* According to the Spanish National Institute of Statistics data (INE), the capital income share in Spanish GDP was 0.4846 in 2018. Consequently, we choose $\theta =0.4846$.

To determine the value of the interest rate, we proceed as follows. According to the INE, Corporate Profit Tax collections amounted 29,711 million euros in 2018, or 2.24 percent of GDP at market prices. Then,

32 $$\begin{aligned} 0.0224= \tau _{k} r \frac{K}{Y^{*}} \end{aligned}$$

According to the BBVA database, in 2016 the value of the Spanish capital stock was 3,281,631 million euros.^15^ According to the INE in 2016 the Spanish Gross Domestic Product at market prices was 1,113,840 million euros. Dividing these two numbers, we obtain $K/Y^{*}=2.94$. We already set $\tau _{k}=0.25$. Consequently, the value for the interest rate is $r=3.0476\%$, which is our target value for the model economy interest rate.

Finally, we obtain the value for the depreciation rate, $\delta$. According to the firm’s optimality conditions:

33 $$\begin{aligned} r=\theta \frac{Y}{K} - \delta \end{aligned}$$

where *Y* is output at factor cost. According to the INE, in Spain in 2018 this number was 977,345. Consequently, the value for the depreciation rate is $\delta =0.1138$.

*Normalizations* Finally, in our model economy there are four normalization conditions. The transition probability matrix on the stochastic component of the endowment of efficiency labor units process is a Markov matrix, and therefore, its rows must add up to one. This property imposes three normalization conditions. We also normalize the first realization of this process to be z(1) = 1.

*Adding up* So far we have determined the values of 36 parameters either directly or as functions of our guesses for (*Y*, *L*) only. We report their values in Table 19.

#### A.3.2 Parameters determined solving a system of equations

We still have to determine the values of 6 parameters. To find the values of those 6 parameters we need 6 equations, where these 6 equations require that model economy statistics replicate the value of the corresponding statistics for the Spanish economy in 2018.

**Table 20:** Macroeconomic aggregates and ratios in 2018 (%)

	$P/Y^{*a}$	$U/Y^{*b}$	$T_{r}/Y^{*}$	$GW^{c}$	$W^{d}$	$I^{e}$
Spain	10.47	1.32	0.83	0.67	59.59	5.16

$^{a}$Variable $Y^{*}$ denotes GDP at market prices.

$^{b}$The ratio $U/Y^{*}$ is the Unemployment benefits as a share of Output at market prices.

$^{c}$Variable *GW* is the Gini Index of wealth.

$^{d}$Variable *W* is the share of workers in the Spanish population with 20+ years old.

$^{e}$Variable *I* is the share of inactive in the Spanish population with 20+ years old

*Aggregate targets* According to the INE, unemployment benefits amounted 17,469 million. That same year, and according to the Spanish Instituto Nacional de la Seguridad Social, pension payments were 125,899 million euros. Finally, and according to Cañón et al. (2016), the sum of different subsidies aimed to protect those people who do not receive any public benefit amounted 8,976 million euros in 2015.^16^ Consequently, the ratios of these variables to GDP at market prices are 1.32, 10.47 and 0.83 percent.

According to Anghel et al. (2018), the Gini Index of wealth in 2014 in Spain was 0.67. Finally, and according to the Encuesta de Población Activa (INE), in Spain in 2018 there were 32,433,800 people aged 20+ years old.^17^ That same survey reports that 19,327,700 were workers and 3,479,100 were unemployed. Consequently, these numbers imply that the share of workers was 59.59 percent and the share of unemployed were 10.72 percent.

*The parameters* The 6 parameters determined by the system are the following:

- Preferences: $$\beta $$, $$\alpha $$, and $$\gamma $$.
- Pension system: $$p_{r}$$.
- Fiscal policy: $b_{0}$, and $\overline{t_{r}}$.

To solve for these values, we follow the procedure proposed by Castañeda et al. (2003) by using a standard minimization routine. Specifically, we use a modification of Powell’s hybrid method, implemented in subroutine DNSQ from the SLATEC package. The DNSQ routine works as follows:

1. Choose the weights that define the loss function that has to be minimized
2. Choose a vector of initial values for the unknown parameters
3. Solve the model economy
4. Update the vector of parameters
5. Iterate until no further improvements of the loss function can be found

Table 21 provides the values for the 6 parameters.

**Table 21:** Parameters, values, and targets

Parameter	Value	Target
$\beta$	0.9965	*GW*
$\alpha$	$28\times 10^{4}$	*W*
$\gamma$	1.7281	*I*
$p_{r}$	0.7650	$P/Y^{*}$
$\overline{t_{r}}$	0.8300	$T_{r}/Y^{*}$
$b_{0}$	0.3518	$U/Y^{*}$

## B. The benchmark economy without unemployment insurance and backpack: open economy

Compared with the baseline economy, replacing the existing unemployment benefit system with a backpack system (BP) with a contribution rate of 6.0% has a large impact in the economy. Households enter the economy without backpack claims. Absent an unemployment insurance policy, young households need to privately insure the risk of losing a job early in life. Aggregate savings increase substantially, more than 40% relative to the baseline economy.

**Table 22:** Aggregates and output ratios in the baseline economy and in the economy without UI and BP (No UI nor BP), and with BP system (Only BP)

Values					
	Baseline	No Ui nor BP	$\%\Delta$	Only BP	$\Delta$
Y	2.1595	2.1157	$-$2.00	2.1318	$-$1.29
A	3.9348	4.5302	15.13	5.5248	40.40
L	0.6912	0.6771	$-$2.00	0.6823	$-$1.29
H	0.1936	0.1904	$-$1.66	0.1921	$-$1.65
C	0.7822	0.7647	$-$2.24	0.7901	1.00
G	0.4114	0.4114	0.00	0.4114	0.00
I	0.8258	0.8090	$-$2.00	0.8151	$-$1.29
w	1.6102	1.6102	0.00	1.6102	0.00
r ($\%$)	3.0476	3.0476	0.00	3.0476	0.00

*Y*: output at factor cost, *A*: total assets, *L*: labor in efficiency units, *H* hours of labor supply, *C*: aggregate private consumption plus consumption tax collections, *G*: government consumption, *I*: investment, *w*: wage rate, *r*: interest rate. The ratio *K*/*Y* measured in model units and not in percentage terms.

$^{a}:$ Difference in percentage points.

${*}:$ As a share of output at factor cost

As Table 23b shows, low productivity workers are hurt the most. The negative welfare effects are almost the same as if the unemployment system is eliminated and there is no ‘backpack’  to replace it, as Table 23a shows.

The introduction of a backpack system with a rate of 6$\%$, in an economy without unemployment insurance, slightly increases the welfare losses of households, compared to the Benchmark economy. Note that in an economy without unemployment insurance, households increase their savings rates, especially for precautionary reasons. Consequently, the introduction of the backpack system, with a high rate, reduces welfare for those liquid constrained households.

**Table 23:** Consumption equivalent variation (%) in average lifetime utility relative to the baseline economy, in an alternative scenario:

Education	Labor productivity		
	Low	Medium	High
(a) Without unemployment insurance			
Dropouts	$-$9.13	$-$5.78	$-$2.79
High School	$-$10.83	$-$6.86	$-$3.34
College	$-$15.01	$-$9.77	$-$5.40
(b) With only a backpack system			
Dropouts	$-$9.24	$-$5.44	$-$1.32
High School	$-$11.15	$-$6.89	$-$2.48
College	$-$11.76	$-$10.63	$-$5.80

## C. More on the closed economy simulation

To calibrate Spain as a closed economy, we implement three main changes with respect to the procedure detailed in Appendix A: we change the value for the discount factor, $\beta$, for the depreciation rate, $\delta$, and for the capital income tax rate $\tau _{k}$.

$\beta$: to replicate the Spanish capital to output ratio. According to the BBVA database, in 2016 the value of the Spanish capital stock was 3,281,631 million euros. According to the *Instituto Nacional de Estadística* (INE) in 2016 the Spanish Gross Domestic Product at market prices was 1,113,840 million euros. Dividing these two numbers, we obtain $K/Y=2.94$, which is our target value for the model economy capital to output ratio. This implies a value of $\beta = 0.9915$.
$\delta$: to replicate the Spanish domestic consumption to output ratio. According to the INE, Private Consumption plus indirect taxes was 654,574 million euros in 2018. Consequently, the ratio of this variable to output at market prices is 54.35 percent. This implies a value of $\delta = 0.0858$.
$\tau _{k}$: to replicate the output share of Corporate Profit Taxes. According to the INE, in 2018, the Corporate Profit Tax collected 29,711 million euros, 2.24 percent of output at market prices. This implies a value of $\tau _{k}=0.1188$.

## D. The benchmark economy without unemployment insurance and backpack: closed economy

**Table 24:** Aggregates and output ratios in the baseline (closed) economy and in the economy without UI and BP (No UI nor BP), and with BP system (Only BP)

Values					
	Baseline	No Ui nor BP	$\%\Delta$	Only BP	$\Delta$
Y	2.0419	2.0473	0.26	2.6166	28.14
K	6.5850	6.7941	3.17	11.1305	69.02
L	0.6785	0.6632	-2.24	0.6712	$-$1.08
H	0.1911	0.1870	-2.15	0.1892	$-$1.00
C	0.9459	0.9348	-1.18	1.0919	15.43
G	0.3891	0.3891	0.00	0.3891	0.00
I	0.7068	0.7234	2.34	1.1356	60.66
w	1.5495	1.5922	2.75	2.0109	
r ($\%$)	6.4441	6.0160	-6.65	2.8059	

*Y*: output at factor cost, *K*: capital stock, *L*: labor in efficiency units, *H* hours of labor supply, *C*: aggregate private consumption plus consumption tax collections, *G*: government consumption, *I*: investment, *w*: wage rate, *r*: interest rate. The ratio *K*/*Y* measured in model units and not in percentage terms.

$^{a}:$ Difference in percentage points.

${*}:$ As a share of output at factor cost

**Table 25:** Consumption equivalent variation (%) in average lifetime utility relative to the baseline (closed) economy, in an alternative scenario:

Education	Labor productivity		
	Low	Medium	High
Without unemployment insurance			
Dropouts	$-$6.23	$-$3.88	$-$2.39
High School	$-$7.79	$-$4.60	$-$2.40
College	$-$12.13	$-$7.06	$-$3.57
With only a backpack system			
Dropouts	7.11	6.45	2.81
High School	6.04	7.28	4.03
College	2.92	6.33	5.71

## E. A closer look at *life cycle consumption profiles*

We further investigated the reasons behind the abrupt jump in consumption profiles around the average retirement age shown in Figs. 3 and 7.

Specifically, we analyze the change in average consumption and net total income between ages 61 and 62 (the first retirement age), for agents who choose to retire, and for different labor market groups, in the two economies (baseline PAYG and BP reformed, both open economies).

### Definitions

To this end, we define net income as the sum of:

1. Net earnings: labor earnings, net of payroll taxes, income taxes, and the BP tax rate (only for the PAYG+BP economy)
2. Net capital income: $(1-\tau _{k}) \, r\, a$
3. Unemployment benefits: recall that this type of benefit does not pay income taxes.
4. Government transfers: recall that this type of benefit does not pay income taxes.
5. Net PAYG retirement pension: retirement pension, net of income taxes.
6. Net BP annuity (only for the PAYG+BP economy): BP annuity net of income taxes.

Note that we do not consider as net income the change in liquid assets nor the change in BP assets.

### E.1 Findings

1. In both, the PAYG and the PAYG+BP model economies, all unemployed and inactive households choose to retire at the first retirement age 62.
2. This is also the case (in both model economies) for all workers who receive the lower productivity stochastic shock. However, workers with the higher stochastic productivity shocks choose to continue working.
3. The small jump of consumption at retirement in the PAYG economy is mainly driven by the jump in consumption of inactive households (see Table 26). And this is mainly because this group has a substantial increase in net income (when moving from 61 to 62 years), as it starts to collect the PAYG pension (see Table 27).
4. The other groups also collect their pension at age 62, but there is no significant variation in their net income. Finally, there is also a significant increase in the net income of those unemployed at 61 that do not receive unemployment benefits, but this group is small among those aged 61 (and 62).
5. In the reformed PAYG+BP economy, the jump in consumption is because all groups significantly increase their consumption when moving from 61 to 62 years (see Table 26). And the reason why this happens is because all groups significantly increase their net income, since in addition to the PAYG pension, they also collect the BP annuity (see Table 27).
6. Finally, one may ask why people do not smooth consumption between ages 61 and 62. This is because they cannot borrow against future income (PAYG pensions or the BP annuity). In the PAYG+BP economy, there are three insurance systems: UI, PAYG, and BP. Consequently, people save less, and we find that 35 percent of those aged 61 has liquid assets less than 10 percent of per capita GDP. Recall that this number is 7 percent in the PAYG Economy (the Gini of Wealth increases from 0.67 in the PAYG to 0.75 in the PAYG+BP economy). Therefore, savings decrease significantly but there is no possibility of borrowing to allow to smooth the consumption profile over time.

In sum, the noted ‘distortion into agents’ intertemporal consumption plans is an exacerbation of the distortion of the PAYG system in establishing a *minimum retirement age*, while it is not possible to borrow from after-retirement income: the pension PAYG economy and the pension plus the accumulated backpack assets in the economy with the BP, which explains the higher jump in consumption in the latter.

**Table 26:** Change in average consumption from ages 61 to 62 years old ($\%$)

	Workers 1	Unemp. 1	Unemp. 2	Unemp. 3	Inactive
PAYG					
Dropouts	3.2	5.1	8.8	28.3	17.6
High School	2.1	5.5	8.7	28.9	22.8
College	0.9	8.1	8.0	43.9	31.3
PAYG+BP					
Dropouts	42.9	6.8	9.3	36.5	22.4
High School	34.7	7.6	10.3	45.8	28.6
College	30.8	9.9	15.4	80.4	40.9

Workers 1: workers with shock 1. Unemp. 1: unemployed in their first year collecting unemployment benefits. Unemp. 2: unemployed in their second year collecting unemployment benefits. Unemp. 3: unemployed without unemployment benefits

**Table 27:** Change in average net income from ages 61 to 62 years old ($\%$)

	Workers 1	Unemp. 1	Unemp. 2	Unemp. 3	Inactive
PAYG					
Dropouts	5.8	6.1	7.5	202.3	143.9
High School	0.8	1.8	1.6	287.2	204.9
College	0.4	1.3	0.1	471.8	336.4
PAYG+BP					
Dropouts	34.7	41.1	33.1	248.9	259.2
High School	26.8	33.8	25.3	354.9	343.8
College	26.1	31.9	23.2	586.7	505.9

Workers 1: workers with shock 1. Unemp. 1: unemployed in their first year collecting unemployment benefits. Unemp. 2: unemployed in their second year collecting unemployment benefits. Unemp. 3: unemployed without unemployment benefits

## F The Spanish social security

The Spanish contributory pension system is the most important program of social protection in Spain, where public contributory pensions are provided by the following three programs. First, the *Régimen General de la Seguridad Social* covers the private sector employees and the members of cooperative firms and the employees of most public administrations other than the central governments. Second, the *Regímenes Especiales de la Seguridad Social* cover the self-employed workers and professionals.^18^ And third, the scheme for government employees, or *Régimen de Clases Pasivas* covers public servants employed by the central government and its local branches.

In this article, we focus exclusively on the retirement pensions payed by the *Régimen General de la Seguridad Social*. Consequently, this section describes the key features of this system and its 2011 and 2013 reforms.

*Financing and eligibility* The Régimen General de la Seguridad Social is a mandatory pay-as-you-go scheme. The payroll tax rate is proportional to covered earnings, which are defined as total earnings, excluding payments for overtime work, between a floor and a ceiling that vary by broadly defined professional categories. The payroll tax rate is 28.3 percent, of which 23.6 percent is attributed to the employer and the remaining 4.7 percent to the employee.

Entitlement to an old-age pension requires at least 15 years of contributions. The retirement age that entitles workers to receive a full retirement pension is 65 for workers who have contributed at least 36 years and three months. Previous to the 2011 Pension reform, every worker aged 61 or older could retire earlier paying an early retirement penalty, as long as they had contributed to the pension system for at least 30 years. Exceptionally, workers who had entered the system before 1967 could retire at age 60. The 2011 Reform of the Spanish pension system delayed the early retirement age from 61 to 63 for those workers who decide to retire on a voluntary basis, and it also delayed the full entitlement retirement age from 65 to 67. The delay in the early retirement age was immediate, and the delays in the normal retirement are gradual: one month per year between 2013 and 2018, and two months per year between 2019 and 2027. Consequently, the full entitlement retirement age in Spain will be 66 in 2021 and 67 in 2027.

*Retirement pensions* The main component of the retirement pension is the *Regulatory Base*, defined as the average covered earnings of the last 21 years before retirement. Labor income earned in the last two years prior to retirement enters the calculation in nominal terms, and the covered earnings of the remaining years are re-evaluated using the rate of change of the Spanish Consumer Price Index. The 2011 Reform of the Spanish pension system extended the number of years of earnings used by the Regulatory Base up to the last 25 years before retirement. The extension of the number of years used to compute the pensions was phased out gradually and it will end in 2022. In addition, the Regulatory Base in multiply by a percentage which depends on the age of the retirees and on the number of years of contributions. And, each year worked after the full entitlement retirement age increases the Regulatory Base in 2 or 3 percentage points depending on the length of the contributory career. Finally, retirement pensions are bound by a minimum and a maximum pension, where minimum pensions depend the pensioner’s age and on the composition of the household.

*The revaluation of pensions* In 2018, the Spanish pension system returned to a full price indexation of pensions.^19^

*The pension reserve fund* Since 2000, part of the surpluses generated by the pension system are deposited in a Pension Reserve Fund. However, and since the stock of assets of this fund only represented 0.4 percent of GDP at the end of 2018, which is our calibration target year, we assume that there is no Pension Fund in our model economy.

## G. Definition of a stationary equilibrium in the BP economy

Let $j\!\in \!J$, $h\!\in \!H$, $z\!\in \!{\mathcal {Z}}$, $s\!\in \!{\mathcal {S}}$, $a\!\in \!A$, and $b\!\in \!B$ and let $\mu _{j,h,z,s,a,b}$ be a probability measure defined on $\Re = J \!\times \! H \times \! {\mathcal {Z}} \!\times \! {\mathcal {S}}\!\times \! A \!\times \! B$.^20^ Then, a stationary competitive equilibrium for this economy is a government policy, $\{G,P,T_{r},U,T_{k},T_{s},T_{y},T_{c},E\}$, a household policy, $\{c(j,h,z,s,a,b)$, *l*(*j*, *h*, *z*, *s*, *a*, *b*), *e*(*j*, *h*, *z*, *s*, *a*, *b*), *r*(*j*, *h*, *z*, *s*, *a*, *b*), $a^{'}(j,h,z,s,a,b)$, $b^{'}(j,h,z,s,a,b) \}$, a measure, $\mu$, factor prices, $\{r, w\}$, macroeconomic aggregates, $\{C$,*Y*,*K*,$L\}$, and a function, *Q*, such that:

*(i)*: The government policy satisfy the consolidated government described in Expressions (10)–(11).
*(ii)*: Firms behave as competitive maximizers. That is, their decisions imply that factor prices are factor marginal productivities $r=f_1\left( K,L\right) -\delta$ and $\omega =f_2\left( K,L\right)$.
*(iii)*: Given the government policy, and factor prices, the household policy solves the households’ decision problem defined in Expressions (15), through (30).
*(iv)*: The stock of assets, consumption, the aggregate labor input, pension payments, unemployment benefit payments, lump sum transfers, tax revenues, and accidental bequests are obtained aggregating over the model economy households as follows: $$\begin{aligned} A= & {} \int \; a + b \; d\mu \\ C= & {} \int \; c \; d\mu \\ L= & {} \int \; \epsilon _{jh} z l \; d\mu \\ U= & {} \int \; ub \; d\mu \\ T_{r}= & {} \int \; t_{r} \; d\mu \\ T_{c}= & {} \int \; \tau _{c} c \; d\mu \\ T_{k}= & {} \int \; \tau _{k}ra \; d\mu \\ T_{p}= & {} \int \; \tau _{p} y \; d\mu \\ T_{y}= & {} \int \; \tau _{y} {\hat{y}} \; d\mu \\ E= & {} \int \; (1-\psi _{j})(1+r) a^{'} \; d\mu \end{aligned}$$ where all the integrals are defined over the state space $\Re$.
*(vi)*: The goods market clears: 34 $$\begin{aligned} C + \int (a^{'}+b^{'}-(1-\delta )(a+b))d \mu +G+NX= F(K,L). \end{aligned}$$ where the last term of the left-hand side of this expression, *NX*, are net exports.
*(vii)*: The law of motion for $\mu _{j}$ is: 35 $$\begin{aligned} \mu _{j+1}=\int _{\Re } Q d\mu _{j}. \end{aligned}$$

Describing function *Q* formally is complicated because it specifies the transitions of the measure of households along its five dimensions: age, education level, productivity, employment status, and assets holdings. An informal description of this function is the following: We assume that new entrants, who are 20 years old, enter to the economy as workers, unemployed, or inactive, following the shares of these groups for the 20-24 cohort in the Spanish economy in 2018, and that they own zero assets. Moreover, workers enter the economy with a job opportunity, that they draw the stochastic component of their endowment of efficiency labor units from its invariant distribution. Their educational shares are exogenous. The evolution of $\mu _{jh}$ is exogenous, it replicates the distribution by age and education of the Spanish population in our calibration target year, 2018. The evolution of $\mu _{z}$ is governed by the conditional transition probability matrix of its stochastic component. The evolution of $\mu _{s}$, is governed by the exogenous probabilities of find/loss a job, by the endogenous employment and search decisions, and by the optimal decision to retire. The evolution of $\mu _{a}$ is determined by the optimal savings decision and by the changes in the population. The evolution of $\mu _{b}$ is determined by the backpack law of motion. The evolution of $\mu _{d}$ is given by the deterministic evolution of unemployment spell duration.

### G.1 Steady-state dynamics

The steady-state dynamics of the economies under study have the following characterization. Given a distribution of households entering the economy ($j=20$ and $a=0$; say, at *T*) they all receive a job opportunity and make their consumption, asset and employment decisions. These households’ decisions together with their survival probabilities define the distribution of this cohort the following year ($T+1$) at $j=21$, but it also the distribution of households of $j=21$ at *T*. Similarly, for $j=22,...,100$; that is, the different cohorts coexisting at *T* mirror the evolution of the distribution of households entering the economy at *T* up to the end of their potential survival $j=100$. In other words, the decisions that agents of generation *T* make through their live are already made in the year they enter the labor market by older agents if they have the same state. By construction, this is a steady-state distribution, which is our benchmark distribution. Different economies simply expose the *T* cohort distribution to different public insurance systems, and therefore, all the cohorts coexisting at *T* behave as if the given system was in place when they entered the economy.

## Footnotes

1 For example, Spain had a cap of 8000 euro which in 2021 has been reduced to 2000 euro.

2 See Banco de España (2021); Hijzen and Salvatori (2020) and Conde-Ruiz et al. (2011).

3 We discretize the state space by choosing a finite grid for both liquid assets. However, the optimal choices $a'$ and $b'$ are not constrained to equal a point in the finite grid. Since we store the joint distribution only on the finite grid points for both types of assets, an individual with choice $a' \in (a_{i},a_{i+1})$ is interpreted to choose asset holdings $a_{i}$ with probability *i* and asset holdings $a_{i+1}$ with probability $1-i$, where *i* solves $a' = ia_{i} + (1 - i)a_{i+1}$. We use the same procedure for backpack assets. Finally, we do not restrict optimal choices to lie in the grid by using linear interpolation.

4 Cohort sizes are exogenous and the calibration of cohort sizes will take into account both immigration and fertility

5 Note that this stochastic process does not depend on agents’ labor market state. Assuming instead, as is often done, that productivity (human capital) depreciates during periods of non-employment would exacerbate the costs from a high unemployment rate.

6 *BP* annuity payments and contributions are fully funded at the worker level and therefore do not enter the government or social security budgets.

7 Unless we mention otherwise, our model economies are open economies.

8 Note that any change in $\tau _c$ (and $\tau _p$, for the separate S.S. budget) is an implicit function of the change in $\tau _b$, the BP tax rate; i.e., this is not an exercise in optimal policy in which we optimize over all tax instruments available to the government. The only instrument we experiment with is $\tau _b$ itself. In previous versions of the paper, we experimented with different tax rates adjusting to clear the budget as the BP is introduced, and our policy results are almost unchanged.

9 The effective labor tax, $\tau _e$, is given by $(1-\tau _e)=(1-\tau _y)(1-(\tau _p +\tau _b))/(1+\tau _c)$.

10 Note that Fig. 3 shows total assets held by workers in the economy with the BP, which does not account for the BP stocks held by the insurance companies, or funds, providing annuity insurance, these are also accounted in Table 7; for example, survivors beyond age 90 still receive their annuity from their insurers even if they have zero assets left.

11 See Appendix E for further details on the evolution of consumption close to the retirement age.

12 This jump does not occur in an economy without PAYG, no minimum retirement age and only BP, as the final steady-state economy in Brogueira de Sousa et al. (2021).
